# A Narrative Review of the Expression and Role of Nitric Oxide in Endometriosis

**DOI:** 10.3390/antiox14030247

**Published:** 2025-02-20

**Authors:** Seung Geun Yeo, Yeon Ju Oh, Jae Min Lee, Sung Soo Kim, Dong Choon Park

**Affiliations:** 1 Department of Otorhinolaryngology—Head and Neck Surgery, College of Medicine, Kyung Hee University Medical Center, Kyung Hee University, Seoul 02447, Republic of Korea; yeo2park@gmail.com (S.G.Y.); jmlee3042@khu.ac.kr (J.M.L.); 2 Department of Precision Medicine, Graduate School, Kyung Hee University, Seoul 02447, Republic of Korea; 3 Department of Convergence Medicine, College of Medicine, Kyung Hee University, Seoul 02447, Republic of Korea; 4 Department of Medicine, College of Medicine, Kyung Hee University Medical Center, Seoul 02447, Republic of Korea; 5duswn1203@khu.ac.kr; 5 Department of Biochemistry and Molecular Biology, College of Medicine, Kyung Hee University, Seoul 02447, Republic of Korea; sgskim@khu.ac.kr; 6 Department of Obstetrics and Gynecology, St. Vincent’s Hospital, College of Medicine, The Catholic University of Korea, Seoul 06591, Republic of Korea

**Keywords:** endometriosis, nitric oxide

## Abstract

Nitric oxide (NO) is a key signaling molecule involved in cellular communication and plays a critical role in various biological processes. Given its dual role in the pathogenesis of endometriosis, we conducted a systematic literature review to explore its mechanisms further. Numerous studies have investigated the expression and role of NO in various diseases, including those in the field of gynecology. However, the expression and role of NO in endometriosis remain a topic of ongoing debate. Therefore, we conducted a comprehensive literature review using the Cochrane Library, EMBASE, Google Scholar, PubMed, and SCOPUS databases to evaluate the induction and role of NO in the pathogenesis of endometriosis. Of the 27 papers ultimately reviewed, 22 (81.4%) reported that NO contributes to the pathogenesis of endometriosis, 3 (11.1%) suggested that NO acts as a protective mechanism against endometriosis, and 2 studies (7.4%) found no association between NO and the pathogenesis of endometriosis. The expression and levels of NO in endometriosis were associated with pregnancy, infertility, menstruation, and pelvic pain. Research conducted on rats and mice demonstrated that NO, nNOS, eNOS, and iNOS play significant roles in the development of endometriosis. Most studies suggested that increased NO levels are associated with the pathogenesis of endometriosis.

## 1. Introduction

### 1.1. Endometriosis

Endometriosis is a chronic gynecological condition where tissue similar to the lining of the uterine endometrium starts to grow outside the uterine cavity. This misplaced tissue can be found on the ovaries, fallopian tubes, the outer surface of the uterus, and other organs within the pelvis. Although rare, it can also occur in other parts of the body [1,2]. Endometriosis, a persistent inflammatory disorder, impacts roughly 5–15% of women in their reproductive years and is linked to persistent pelvic discomfort, painful menstruation, pain during intercourse, infertility, and irregular menstrual cycles [3]. While endometriosis is a benign condition, its capacity to penetrate and invade distant tissues resembles the metastatic behavior of malignant tumors [4]. Several hypotheses have been proposed to explain the etiology of endometriosis, such as retrograde menstrual reflux, the presence of endometrial stem cells in ectopic locations, and defects in the immune system [5]. However, the exact pathogenesis of endometriosis has not yet been fully elucidated, but retrograde menstruation remains the closest in this regard. The gold standard diagnostic method is direct observation of the pelvis or biopsy through laparoscopy [6].

The treatment of endometriosis primarily involves pharmacological treatments and surgical interventions. Pharmacological treatments include symptom management and the use of hormonal therapies or aromatase inhibitors to induce amenorrhea or reduce estrogen levels, thereby inhibiting the growth of endometrial tissue. Surgical treatment entails various surgical approaches to excise endometriotic lesions. In cases where other treatments have failed, a hysterectomy may be performed to alleviate symptoms by removing the uterus, thus addressing the root cause of endometriosis. Additionally, alternative and complementary treatments, such as dietary modifications, regular exercise, and stress management, are also utilized [1].

### 1.2. Nitric Oxide

In 1863, Burton first described the therapeutic effects of amyl nitrate on angina pectoris and noted its similarity to the effects of nitric oxide [7]. In 1980, Furchgott and Zawadzki reported that vascular endothelial cells are essential for vascular smooth muscle relaxation in response to acetylcholine, marking a pivotal discovery in endothelial biology [8]. Subsequently, the biological activity of an unstable, diffusible vasodilator was identified to closely resemble that of nitric oxide. In 1981, Mellion first demonstrated that nitric oxide inhibits platelet aggregation [9]. Later, nitroglycerin was found to react with cysteine to form S-nitrosocysteine, which, despite its instability, induces vascular smooth muscle relaxation and vasodilation through the action of released nitric oxide.

In 1985, Stuehr and Marletta first reported the generation of nitrogen oxide NO in mammalian cells [10] and later confirmed that activated murine macrophages produce nitrite (NO_2_^−^) and nitrate (NO_3_^−^). In 1987, Palmer et al. measured nitric oxide using chemiluminescence and demonstrated that the endothelium-derived relaxing factor (EDRF), stimulated by bradykinin in porcine aortic endothelial cells, is predominantly nitric oxide [11]. That same year, the substance generated by endothelial cells responsible for mediating vascular relaxation was conclusively identified as nitric oxide [12]. In 1989, Vallance et al. demonstrated that administration of a nitric oxide synthesis inhibitor in the brachial artery significantly reduced blood flow, underscoring the critical role of nitric oxide in the regulation of vascular relaxation and dilation [13]. Recognizing its significance, the scientific community named nitric oxide the “Molecule of the Year” in *Science* in 1992 [14,15].

Nitric oxide (NO) is a gaseous, inorganic free radical that serves as a signaling molecule mediating diverse physiological processes, including neurotransmission, vasodilation, and host defense. It is synthesized by nitric oxide synthase (NOS) through the metabolism of L-arginine to L-citrulline. Due to its high reactivity and short half-life, NO must be produced locally at its site of action. Direct studies of NO are challenging because of its gaseous state and rapid degradation, leading researchers to focus on NOS activity or its metabolites, nitrite and nitrate, for indirect analysis.

NOS is found in multiple organs and cell types, including macrophages, endothelial cells, platelets, fibroblasts, hepatocytes, and neurons, among others. It is generally classified into three isoforms based on cell type and characteristics: neuronal NOS (nNOS, type I), inducible NOS (iNOS, type II), and endothelial NOS (eNOS, type III) [16,17].

Although toxic as an atmospheric chemical, nitric oxide plays surprisingly beneficial roles in the body. Specifically, NO secreted by vascular endothelial cells not only mediates vasodilation but also inhibits macrophage-mediated cytotoxicity, platelet adhesion, and coagulation. It also facilitates relaxation of the corpus cavernosum, regulates baseline blood pressure, and contributes to synaptic plasticity in neurons. Additionally, NO acts as a key mediator in normal renal and endocrine functions and as a smooth muscle relaxant in pregnancy [18,19,20].

The functions and secretion of NO vary depending on the tissue type, its target, and surrounding physiological conditions. The small amounts of NO produced by vascular endothelial cells regulate the relaxation of adjacent vascular smooth muscle, while large amounts secreted in response to cytokines can exert cytotoxic effects, killing pathogens or, paradoxically, damaging host tissues. Thus, NO acts as an immunomodulator, with effects that are either protective or destructive depending on the local context [21].

## 2. Endometriosis and NO

**Figure 1:**
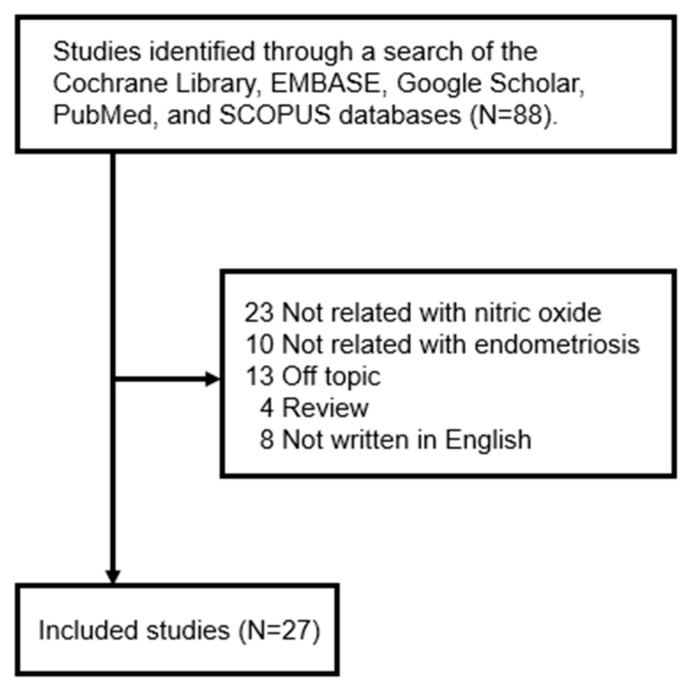
Review flow diagram.

Although various studies have examined the role of NO in different diseases, there has not been a comprehensive review of the literature on the expression and role of NO in the development of endometriosis. While several reviews exist regarding ROS or free radicals and endometriosis, there has not been a paper that specifically reviews experimental data on the impact of NO on endometriosis. Therefore, one author (Y.J.O) searched for studies published between January 1997 and September 2024 in five electronic databases—Cochrane Libraries, EMBASE, Google Scholar, PubMed, and SCOPUS—using the search terms ‘endometriosis’ and ‘nitric oxide’. The literature search focused on studies published in English and included (1) prospective or retrospective studies on NO in endometriosis and (2) studies involving both humans and animals. However, the following were excluded: (1) unpublished data, (2) review articles, (3) gray literature, (4) case reports, and (5) duplicates. Consequently, this study conducted a literature review on 27 out of a total of 88 studies (Figure 1).

The selected studies focused on analyzing the presence or absence of increased NO levels in the context of endometriosis. The papers were categorized based on whether NO played a positive or negative role in the pathogenesis of endometriosis. Among the reviewed studies, 22 reported that NO contributed to the pathogenesis of endometriosis, 3 suggested a protective role of NO against endometriosis, and 2 found no association between NO and the progression or treatment of the condition.

**Figure 2:**
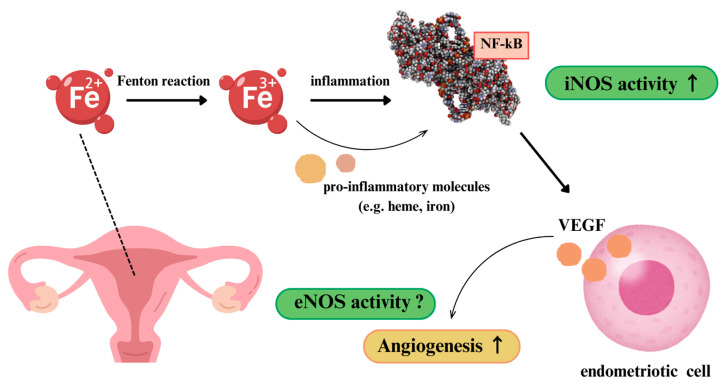
Iron-induced oxidative stress production mechanism related to nitric oxide synthase.

Furthermore, several papers have been found that describe the impact of oxidative stress on endometriosis. According to these studies, the mechanisms by which oxidative stress affects endometriosis occur through the Fenton reaction and inflammation. The Fenton reaction, in particular, is emphasized, as iron-induced oxidative stress plays a key role in the development of endometriosis. In women with endometriosis, iron can accumulate in the peritoneal fluid, macrophages, and endometrial lesions. This excess iron can lead to the production of harmful free radical species via the Fenton reaction, resulting in oxidative stress. Additionally, proinflammatory molecules, such as heme and iron, generate several transcription factors and activate NF-kB. This NF-kB factor induces several molecules, including IL-1 (interleukin-1), IL-6, IL-8, TNF-α (tumor necrosis factor-alpha), and particularly iNOS. NF-kB also appears to play a role in the angiogenesis of endometriotic cells. Oxidative stress can enhance VEGF production, which can stimulate angiogenesis on its own. Furthermore, eNOS promotes angiogenesis [22]. Thus, iron-induced oxidative stress may potentially be related to the production of iNOS and eNOS (Figure 2).

### 2.1. Studies Suggesting That Increased NO Contributes to the Pathogenesis of Endometriosis (Table 1)

#### 2.1.1. Studies Reporting Increased NO Levels in Endometriosis

Studies suggesting an association between increased NO levels and the pathophysiology of endometriosis have been consistently reported.

Khorram et al. conducted a comparative study of eNOS protein expression and α_Vβ_3 integrin levels in endometrial biopsy samples from patients with and without endometriosis. Their findings revealed that eNOS expression was significantly increased in the endometrial endothelial cells of patients with endometriosis, whereas no significant changes were observed in the stroma or peritoneal fluid. Furthermore, α_Vβ_3 integrin expression was significantly reduced in the glandular and luminal epithelium of the endometrium in the endometriosis group compared to the controls. These results suggest that nitric oxide plays a critical role in the pathogenesis of endometriosis [23]. Based on these research findings, the upregulation of glandular eNOS may be driven by inflammatory cytokines, which are elevated in endometriosis, and by high local estrogen levels. These factors promote the proliferation of endometriotic implants and further activate eNOS. The increase in local estrogen production may also explain the decreased expression of αvβ3 integrin, as estrogen has been shown to inhibit the expression of this integrin in the endometrial epithelium [24].

Numerous studies have highlighted the role of increased NO in the pathophysiology of endometriosis. Wu MY et al. (2003) reported that iNOS expression is elevated in patients with endometriosis. In a comparative analysis of endometrial tissues from 30 patients with myomas and 34 patients with endometriosis, NO levels were significantly higher in ectopic endometrium than in eutopic endometrium. Additionally, iNOS expression was markedly higher in the endometrium of patients with endometriosis compared to controls [25].

Similarly, Maryam Kianpour (2015) compared serum and peritoneal fluid (PF) levels of NO metabolites (nitrite), asymmetric dimethylarginine, and estradiol in 90 patients with endometriosis and 89 without. Her findings demonstrated a significant increase in nitrite levels in the PF of patients with endometriosis, suggesting that elevated NO metabolites in PF may contribute to the pathogenesis of endometriosis [26].

Ota H et al. (1998) investigated endometrial cells from 35 patients with endometriosis, 33 with adenomyosis, and 46 fertile controls. Their study revealed that in fertile women, eNOS expression in the surface and glandular epithelium exhibited cyclical changes, being lowest during the early proliferative phase, increasing progressively to peak in the mid-secretory phase, and then declining. In stromal cells, no such cyclical changes were observed. However, in patients with endometriosis and adenomyosis, eNOS expression remained persistently higher than control levels throughout the menstrual cycle. This suggests a pathological role for eNOS overexpression in these conditions [27].

Barbara H. Osborn (2001) compared peritoneal fluid and macrophages from nine infertile women with endometriosis and nine fertile controls who underwent laparoscopy. Her study showed higher NOS enzyme activity in macrophages from the patients with endometriosis. Immunoblot analysis revealed NOS_2_ protein expression exclusively in the macrophages of the patients with endometriosis. While NO concentrations in PF were similar between the two groups, the total PF NO content was higher in the patients with endometriosis. After three days of in vitro culture, macrophages from the patients with endometriosis produced significantly more NO in response to IFN-α or IFN-γ and LPS than those from the controls. Elevated NO levels may negatively affect sperm, embryos, implantation, and tubal function, suggesting that reducing PF NO production or blocking its effects could improve fertility in women with endometriosis [28].

Wu MY et al. also demonstrated that in patients with advanced stages of endometriosis, levels of total antioxidants and NO were significantly higher than in those with early-stage endometriosis [29].

Similarly, in a prospective study, M.G. Rocha et al. found increased NO levels in women with chronic pelvic pain due to endometriosis. NO levels were directly correlated with a reduction in pain intensity and increased pain thresholds after treatment, demonstrating a significant association between NO levels and endometriosis-related pain [correlation = 0.67 (95% CI = 0.35–0.85), *p* < 0.0001] [30].

Several studies also suggested a link between elevated NO levels and infertility in endometriosis. Abhay K. Singha et al. compared follicular fluid (FF) samples from 200 patients with endometriosis and 140 patients with tubal infertility. NO concentrations were higher in patients with endometriosis than in those with tubal infertility. Additionally, pregnant women showed significantly lower levels of NO, ROS, and MDA compared to non-pregnant endometriosis cases (*p* < 0.001), indicating that elevated NO and ROS levels may impair oocyte and embryo quality in endometriosis and tubal infertility [31].

Qiong Luo et al. (2010) analyzed PF from 82 women with infertility, including 44 with minimal and 40 with mild endometriosis, and 20 controls. Their findings suggested that increased NO levels negatively impacted oocyte fertilization and preimplantation embryo development, highlighting a potential role of NO in the pathogenesis of endometriosis-associated infertility [32].

Pravin T. Goud et al. compared FF, granulosa cells (GCs), immature oocytes (IOs), and ART outcomes between women with and without endometriosis. Women with endometriosis had significantly lower peak serum E2 levels, higher apoptosis and nitrotyrosine staining in GCs, and increased rates of cortical granule loss, spindle disruption, and zona pellucida dissolution in in vitro matured oocytes. FF nitrate levels were significantly higher in non-pregnant patients with endometriosis compared to pregnant ones. These findings suggest that altered follicular environments and poor oocyte quality in endometriosis are influenced by oxidative dysregulation of NO [33].

Endometriosis is characterized by repeated inflammatory changes and severe adhesions, inducing both innate and adaptive immune responses in the peritoneal cavity. Seung Geun Yeo et al. analyzed peritoneal effusions from 40 patients with endometriosis and 40 controls, focusing on Toll-like receptors (TLR-1, -2, -4, -5, and -9), nucleotide-binding oligomerization domains (NOD-1 and -2), interleukins (IL-1β, -6, -8, -10, and -12), interferon-γ, tumor necrosis factor-α, CA 125, iNOS, eNOS, and immunoglobulins (Igs). They found significantly higher levels of TLR-2 and -9, NOD-1 and -2, iNOS and eNOS mRNA, and CA 125 in the endometriosis group compared to the controls (*p* < 0.05) [34].

#### 2.1.2. Studies on Polymorphisms Related to NO in Endometriosis

According to the study by Kim Hoon et al., altered expression of eNOS has been associated with the development of endometriosis. The genotype of the eNOS gene (NOS3) is implicated not only in variations in enzyme activity but also in changes in plasma NO levels. Kim’s research indicated that the Glu298Asp polymorphism of NOS3 might regulate angiogenesis and influence individual susceptibility to endometriosis. In their study involving 299 women with endometriosis and 459 controls without the condition, genotypic analysis of the Glu298Asp polymorphism revealed that the frequency of the non-GG genotype (GT + TT) was significantly higher in the endometriosis group compared to the controls (*p* = 0.001). These findings suggest that the T allele, encoding aspartic acid in the Glu298Asp polymorphism of NOS3, may be associated with advanced stages of endometriosis [35].

Similarly, Sevasti Zervou (2003) investigated the Glu298Asp mutation in blood samples from 94 patients with endometriosis and 60 controls. Their results showed that the frequencies of heterozygous genotypes and the Glu298Asp genotype were significantly higher in the endometriosis group compared to the controls. Furthermore, the frequency of the mutant T allele was also elevated in the endometriosis group. Notably, the presence of the T allele was associated with a ten-fold increased risk of developing endometriosis in the studied population. These findings propose that genetic variations in the eNOS gene may contribute to aberrant angiogenesis in the endometrium and impairments in the development and function of the human reproductive system [36].

#### 2.1.3. Studies Demonstrating Symptom Improvement or Therapeutic Effects in Endometriosis via NO Inhibitor Use

Machado DE et al. (2023) investigated the effects of clotrimazole (CTZ) on endometriosis in a rodent model. Eighteen rats underwent autologous endometrial implantation and were randomized into two groups, with one group receiving 200 mg/kg of CTZ daily for 15 days. Compared to the controls, the CTZ group showed significant reductions in lesion growth, implant size, glandular atrophy, serum NO levels, macrophage count, and iNOS immunoreactivity. Additionally, CTZ decreased lipid peroxidation and protein carbonylation in the liver, as well as superoxide dismutase (SOD) activity, while increasing glutathione S-transferase (GST) activity. These findings suggest that CTZ promotes the regression and atrophy of endometriotic lesions by downregulating iNOS expression, reducing reactive nitrogen species (RNS) production, and enhancing the antioxidant system [37].

Cayci T et al. (2011) assessed the effects of infliximab, etanercept, and letrozole on 41 rats with experimentally induced endometriosis. Plasma asymmetric dimethylarginine (ADMA) levels were elevated, while NOx levels were decreased in the treatment groups compared to the controls. The reduction in plasma NOx levels correlated with the regression of endometriosis, underscoring the potential role of NO modulation in disease progression [38].

Wang XL et al. (2008) evaluated the anti-inflammatory effects of a selective ER-beta (ERβ) agonist on lipopolysaccharide (LPS)-induced iNOS expression in peritoneal macrophages (PMs) from patients with endometriosis. The study found that PMs from the patients with endometriosis expressed higher levels of ERβ compared to the controls. Pre-treatment with ERB-041 significantly inhibited LPS-induced iNOS expression and NF-κB activation, highlighting the therapeutic potential of ERβ agonists in modulating inflammatory pathways in endometriosis [39].

DLBS1442, an active compound extracted from Phaleria macrocarpa, was studied by Olivia M Tandrasasmita. Applying DLBS1442 to human endometrial RL95-2 cell lines for 24 h showed dose-dependent inhibition of angiogenesis and cell migration. At 100 μg/mL, the sub-G1 cell proportion increased from 7% to 34%, indicating enhanced apoptosis. DLBS1442 also decreased estrogen receptor levels, increased progesterone receptor levels, and suppressed the eicosanoid pathway by downregulating NFκB transcription and iNOS expression. These findings suggest DLBS1442 as a promising agent for alleviating endometriosis symptoms through anti-angiogenic, anti-inflammatory, and pro-apoptotic mechanisms [40].

Noscapine, a natural alkaloid with anti-angiogenic properties, was tested by Mohammad Rasool Khazaei et al. (2018) using a 3D culture model of patients with eutopic endometrium from endometriosis (EEE) and normal endometrium (NE). Noscapine significantly inhibited growth in both the EEE and NE groups in a dose-dependent manner (0–200 μM). Apoptosis-related gene expression was increased, while Bcl-2 and Sirt1 levels were reduced. Notably, NO secretion was significantly decreased in both groups, emphasizing the role of NO reduction in the therapeutic effects of noscapine for endometriosis [41].

Daniel Escorsim Machado et al. (2023) highlighted CTZ’s efficacy in reducing endometriotic lesion growth, implant size, and inflammation markers in rats, corroborating its potential as an endometriosis therapy [42].

E. Kalehoei et al. (2022) demonstrated that supplementing in vitro maturation (IVM) media with L-carnitine (LC) and bone marrow mesenchymal stem cell-conditioned medium (BMSC-CM) improved blastocyst development and reduced nitro-oxidative stress in an EMS-induced mouse model. LC and BMSC-CM supplementation increased the total antioxidant capacity (TAC) and moderated NO levels, suggesting their utility in enhancing oocyte quality and preimplantation development [43].

Dan Wang et al. (2018) studied the effects of 6-shogaol, a bioactive compound, on NF-κB signaling and inflammation in a rat model of endometriosis. Oral administration of 6-shogaol (50–150 mg/kg) for one month significantly downregulated NF-κB activation, VEGF, and VEGFR-2 expression while reducing pro-inflammatory cytokines (IL-1β, IL-6), PGE2, and NO levels. These results demonstrate 6-shogaol’s ability to suppress lesion proliferation and regulate COX-2/NF-κB-mediated inflammation [44].

JianHua Wang et al. investigated endometrial eNOS and iNOS expression in 30 women with endometriosis-related infertility and 19 women with carcinoma in situ. Before GnRH-a treatment, eNOS expression was higher in the endometrium of the patients with endometriosis than the controls. After three months of treatment, eNOS levels significantly decreased, correlating positively with serum E2 and P concentrations. These findings suggest that GnRH-a therapy reduces eNOS expression in endometriosis and that ovarian steroid hormones influence eNOS expression in the endometrium [45].

In summary, the production of NO, which influences the pathogenesis of endometriosis, is primarily associated with eNOS and iNOS. For iNOS, it is highly likely that the NF-κB pathway and ERK pathway, both induced by LPS, are involved in its mechanism. Regarding eNOS, it appears to play a significant role in mediating angiogenesis, potentially involving VEGF. The pathogenesis of endometriosis is generally associated with inflammatory processes, and in addition to NO, substances such as IL-1, IL-6, PGE2, COX-2, and SOD have been observed. Therefore, immune responses, including those by peritoneal macrophages, seem to significantly contribute to the pathogenesis. The potential influence of hormones, such as estrogen or progesterone, cannot be ruled out either.

**Table 1:** Studies suggesting that increased NO contributes to the pathogenesis of endometriosis.

Author [Reference]	Study Design	Subjects	Sample(s)	Detection Method	Target Pathway (s) Associate with NOS	Results/Conclusions
**Khorram O, et al. (2002) [23]**	human study	9 fertile women and 30 infertile women with endometriosis. 13 women without and 12 women with endometriosis	endometrial tissue, peritoneal fluid	immunohistochemical analysis, nitric oxide measurement (Sievers Instrument Model 280 Nitric Oxide Analyzer, Inc., Boulder, CO, USA)	eNOS	In patients with endometriosis, eNOS staining was markedly elevated (*p* < 0.001) in the glandular and luminal epithelium compared to controls, while no significant increase was observed in the stroma. In contrast, $\alpha_{V}\beta_{3}$ integrin expression was significantly decreased in glandular (*p* < 0.001) and luminal (*p* < 0.05) epithelium from women with endometriosis than in healthy controls. The NO levels in the peritoneal fluid showed no difference between patients with endometriosis and those without. /Heightened local estrogen synthesis might explain the reduced $\alpha_{V}\beta_{3}$ integrin expression, since estrogen is known to suppress this integrin’s expression in endometrial epithelium. Increased eNOS levels during the embryo implantation window could decrease the endometrium’s receptivity to implantation.
**Wu MY, et al. (2003) [25]**	human study	control tissue from myoma cases (*n* = 30), endometriosis cases (*n* = 34)	endometrial tissue	measurement of nitrite/nitrate (rapid-response chemiluminescence analyser), ELISA study of NOS	eNOS, iNOS	Nitrite/nitrate levels were significantly elevated in women with endometriosis. Although not statistically significant, NO levels appeared higher in ectopic compared to eutopic endometrial tissues. Additionally, there was a modest rise in endometrial iNOS protein levels in women with endometriosis compared to those without. /Increased levels of NO and NOS in the endometrial tissues of women with endometriosis suggest a potential involvement of NO in the disease’s pathogenesis.
**Kianpour M, et al. (2015) [26]**	human study	women with endometriosis (*n* = 90), without endometriosis (*n* = 89)	serum, peritoneal fluid	ELISA study, Griess method	NO	The estradiol concentration in peritoneal fluid (PF) was notably higher than in the serum. Both nitrite and ADMA levels were significantly elevated in the serum compared to the PF in both groups. However, nitrite levels in the PF were significantly higher in patients with endometriosis compared to those without the condition. Additionally, there were no significant differences in serum ADMA and nitrite levels, nor in PF ADMA levels, between the two groups. /The NO metabolite levels in the peritoneal fluid suggest a potential role of NO in the pathogenesis of endometriosis.
**Ota H, et al. (1998) [27]**	human study	35 patients with endometriosis, 33 patients with adenomyosis, and 46 fertile controls	endometrial tissue	semiquantitative immunostaining, histologic examination	eNOS	Analyses showed phase-dependent variations in endothelial nitric oxide synthase expression in the surface and glandular epithelia during the menstrual cycle in fertile controls. Expression was minimal in the early proliferative phase, increased gradually, peaked in the midsecretory phase, and declined afterward. In contrast, stromal cell expression remained constant throughout the cycle. Unexpectedly, in endometriosis and adenomyosis, endothelial nitric oxide synthase expression consistently exceeded control levels throughout the menstrual cycle. /This study demonstrated phase-dependent changes in endothelial nitric oxide synthase during the menstrual cycle. The heightened expression of this enzyme in the endometrium throughout the cycle suggests a potential pathological role in endometriosis and adenomyosis.
**Osborn BH, et al. (2001) [28]**	human study	9 infertile women with endometriosis and 9 fertile woman without endometriosis	peritoneal macrophage, peritoneal fluid	NOS enzyme activity, NOS2 protein expression by immunoblot, and nitrite/nitrate assay	iNOS (NOS2)	NOS enzyme activity was elevated in peritoneal macrophages from patients with endometriosis, with immunoblots showing NOS2 protein only in these patients’ macrophages. While peritoneal fluid NO concentration was similar between groups, total NO content was higher in patients with endometriosis. After 3 days of culture, peritoneal macrophages from these patients produced more NO in response to IFN-α or IFN-γ plus LPS compared to controls. /Peritoneal macrophages from women with endometriosis-associated infertility exhibit elevated NOS2 expression, increased NOS enzyme activity, and greater NO production in response to immune stimulation in vitro. Since high NO levels can negatively impact sperm, embryos, implantation, and oviductal function, reducing NO production in peritoneal fluid or blocking its effects may enhance fertility in these women.
**Wu MY, et al. (1999) [29]**	human study	women with endometriosis (early, *n* = 12; advanced, *n* = 11), without endometriosis (*n* = 13)	peritoneal macrophage culture	ELISA study, Griess method	NO	Following 24-h stimulation with 2 ng/mL LPS, peritoneal macrophages from women with early-stage endometriosis secreted more NO, IL-6, and IL-10 than controls. Women with advanced endometriosis exhibited higher IL-12 levels compared to controls. Additionally, after LPS stimulation, the advanced endometriosis group showed greater total antioxidant levels than the early-stage group. /After LPS stimulation, PMs yielded more NO in the early stage of endometriosis. Further clarification is needed to determine if the elevated IL-10 causes these peritoneal macrophages from patients with endometriosis to remain temporarily inactive and, subsequently, release more NO and total antioxidants following in vitro LPS stimulation.
**Rocha MG, et al. (2015) [30]**	human study	25 healthy women, 40 women with chronic pelvic pain (endometriosis group, *n* = 24; myofascial pain syndrome, *n* = 16)	plasma sample	pain measurement using a visual analogue scale (VAS), NO measurement using the colorimetric Griess method	NO	In the endometriosis group, reduced pain intensity correlated with decreased NO levels [correlation = 0.67, 95% CI = 0.35 to 0.85, *p* < 0.0001]. Additionally, a reduction in NO levels was linked to an increased pain threshold [correlation = –0.53, 95% CI = –0.78 to –0.14, *p* < 0.0001]. /In women with chronic pelvic pain due to endometriosis, NO levels were elevated and directly linked to a reduction in pain intensity and an increase in pain threshold following treatment.
**Singh AK, et al. (2012) [31]**	human study	340 infertile women (200 women with endometriosis, 140 with tubal infertility without endometriosis)	follicular fluid	chemiluminescence assay, Griess reaction, thiobarbituric acid method, spectrophotometric method, HPLC	NO	Patients with endometriosis exhibited a significant increase in NO expression compared to controls. The concentration of NO was found to be significantly higher in follicular fluid corresponding to immature oocytes and poor-quality embryos. Pregnant women exhibited significantly lower levels of NO, ROS, and MDA compared to non-pregnant endometriosis cases (*p* < 0.001). /Compared to tubal infertility, endometriosis showed increased NO concentrations. Elevated ROS and NO levels in both endometriosis and tubal infertility were linked to poor oocyte and embryo quality. Additionally, higher levels of ROS and NO were observed in women who did not conceive compared to those who did.
**Qiong Luo (2010) [32]**	human study	84 women with endometriosis, 100 women with tubular block, 20 control women groups	peritoneal fluid	fertilization rate, cleavage rate, fertilization rate (all indexes were recorded when IVF-ET were conducted)	NO	Oocyte fertilization rate of women with endometriosis with IVF-ET treatment was significantly lower than that of women with tubal blocks. The dose-related adverse effects of endometriotic PF and SNP (NO donor) in culture medium on oocyte fertilization and embryos development were confirmed. /Elevated NO levels in peritoneal fluid are crucial in mediating the impact of endometriotic fluid on oocyte fertilization and embryo development.
**Goud PT et al. (2014) [33]**	human study	patients with endometriosis (*n* = 10), without endometriosis (*n* = 18)	follicular fluid, granulosa cells, oocytes	TUNEL assay, follicular fluid nitrate/nitrite colorimetric assay	NO	Clinical characteristics and ART live birth outcomes were similar between groups A (with endometriosis) and B (without endometriosis). However, women in group A had significantly lower peak serum E2 levels, and higher apoptosis and nitrotyrosine staining in granulosa cells compared to group B. Group A also showed fewer immature oocytes maturing to MII stage. IVM oocytes in group A exhibited a higher incidence of cortical granule loss, spindle disruption, and longer zona pellucida dissolution timing. Additionally, follicular fluid nitrate levels were significantly higher in women who did not conceive in group A compared to those who did. /Elevated protein nitration, granulosa cell apoptosis, resistance to IVM, and oocyte aging suggest that oxidative dysregulation of NO contributes to an altered follicular environment and poor oocyte quality in women with endometriosis.
**Yeo SG, et al. (2013) [34]**	human study	40 patients with endometriosis, 40 patients with benign tumors	peritoneal effusion	real time PCR	iNOS, eNOS	Levels of iNOS and eNOS mRNAs were significantly elevated in the endometriosis group compared to the non-endometriosis group. /Elevated NOS mRNA expression in peritoneal fluid may be linked to endometriosis.
**Kim H, et al. (2009) [35]**	human study	299 women with advanced stage endometriosis and 459 control women	peripheral blood sample	genotyping of the Glu298Asp polymorphism by PCR-RFLP analysis	eNOS	Genotypic frequencies differed significantly between women with and without endometriosis, with a higher prevalence of the non-GG genotype (GT, TT) in the endometriosis group compared to the control group. /These findings indicate that the T allele, which encodes aspartic acid, of the Glu298Asp polymorphism in NOS3 may be linked to advanced stage endometriosis in the Korean population.
**Zervou S, et al. (2003) [36]**	human study	60 control women and 94 study group women diagnosed with endometriosis	peripheral blood sample	PCR	eNOS	The frequencies of the TG and TT genotypes were found to be significantly higher in the endometriosis group than in the control group. The frequency of the mutant T allele was higher in endometriosis compared with the control group. /Our study showed a 10-fold increased risk of developing endometriosis with the presence of the mutant T allele. These findings support the notion that genetic variations in the endothelial NOS gene might contribute to endometrial angiogenesis and abnormalities in the development and function of human reproductive organs, affecting endometrial cell physiology.
**Machado DE, et al. (2023) [37]**	animal study	18 Wistar rats	endometrial tissue, plasma	hematoxylin and eosin, immunochemistry, morphometric analyses, nitric oxide analysis (Griess reagent, ELISA), flow cytometry, Antioxidant system analysis (SOD, CAT, GST, GSH), lipid peroxidation biomarker analysis	iNOS	In the Clotrimazole (CTZ) group, reductions were observed in lesion growth, implant size, glandular atrophy, serum NO levels, macrophage cell count, and inducible nitric oxide synthase (iNOS) immunoreactivity compared to the control group. /These findings imply that CTZ may inhibit reactive nitrogen species production by reducing iNOS expression, thereby boosting the antioxidant system to induce atrophy and regression of endometriotic lesions, without negatively impacting the brain or liver.
**Cayci T, et al. (2011) [38]**	animal study	55 Sprague–Dawley rats	endometriotic lesion tissue, plasma	plasma ADMA (liquid chromatography), measurement of plasma nitrate/nitrite (Griess reaction), hematoxylin and eosin staining	NO	Forty-one rats with endometriotic implants were divided into four groups and treated with infliximab, etanercept, letrozole, or given no treatment (control), respectively. In groups 1, 2, and 3, plasma ADMA levels were higher compared to the control and normal groups at 296.8 ± 66.2, 285.9 ± 35.7, 200.3 ± 41.0, 125.3 ± 16.7, and 111.3 ± 6.5 mmol/L, respectively. Conversely, NOx levels were lower than those in the control and normal groups, measuring 19.6 ± 3.8, 19.8 ± 4.4, 39.3 ± 6.1, 80.5 ± 5.3, and 91.1 ± 5.0 mmol/L, respectively. /Infliximab, etanercept and letrozole have regressed endometriotic implants, decreased plasma NO levels, and increased plasma ADMA levels.
**Wang XL, et al. (2008) [39]**	human study	11 women with ovarian endometriomata, 7 women without endometriosis	peritoneal macrophage	real time PCR, Concentration of NO2–NO3 was measured with the NO2−/NO3− assay kit from Cayman Chemicals, immunoblotting	NO, iNOS, ERα, ERβ, NF-κB, ERK pathway	In endometriosis, peritoneal macrophages (PMs) showed higher expression of ERβ compared to those from women without endometriosis. Pretreating PMs with ERB-041 significantly inhibited LPS-induced iNOS expression and NF-κB activation by preventing its nuclear translocation. The ERKs (extracellular signal-regulated kinases) pathway contributed to LPS-induced iNOS production and was not suppressed by ER activation. /The ERβ agonist’s inhibitory effect on LPS-induced iNOS production in EMS PMs is likely mediated through the suppression of the NF-κB signaling pathway, rather than the ERKs pathway.
**Tandrasasmita OM, et al. (2015) [40]**	human cell line study	the human endometrial epithelial cell line (RL95-2)	endometrial epithelial cell culture	RNA analysis (spectrophotometer), gelatin zymography, cell migration scratch assay, in vitro cell toxicity assay, flow cytometry analysis, western blotting	NF-κB, iNOS	Given that NFκB regulates COX-2 and iNOS, and that endometriosis-associated infertility is often linked to elevated iNOS and NOS enzyme activity, we examined DLBS1442’s ability to modulate iNOS expression. The findings show that DLBS1442 suppressed iNOS mRNA expression in RL95-2 cells, likely due to reduced transcriptional levels of NFκB. /Our data suggest that DLBS1442’s anti-inflammatory, analgesic, and anti-angiogenic effects may stem from the downregulation of NFκB activity, leading to reduced expression of iNOS and COX-2.
**Khazaei MR (2018) [41]**	human study	EEE patients (*n* = 8), normal endometrium (NE) were taken from fertile women (*n* = 8)	endometrial tissue	three-dimensional culture (proliferation of epithelial and stromal cells, the angiogenesis and formation of monolayer epithelial cell were evaluated), Nitric Oxide assay (Griess method), real-time PCR	NO	The growth inhibition of eutopic endometrium of endometriosis (EEE) was dose-dependent and endometrial growth was almost completely blocked at 100 and 200 μM doses of noscapine. The growth-inhibitory effect of noscapine was dose-dependent. The test groups showed a time-dependent decrease in NO levels compared to the control group. /NO secretion significantly decreased in both EEE and NE groups. In conclusion, higher doses of noscapine inhibited the growth and angiogenesis in EEE and NE.
**Machado DE, et al. (2016) [42]**	animal study	20 Sprague–Dawley rats	endometrial explant tissue, peritoneal fluid	H&E histology, immunohistochemistry, light microscope, real-time PCR, ELISA immunoassay, flow cytometry, Griess method, cell culture and viability assay	NO	In the CTZ group, there was a significant reduction in lesion growth, implant size, glandular atrophy, serum NO levels, macrophage count, and inducible nitric oxide synthase (iNOS) immunoreactivity compared to the control. CTZ (*p* < 0.05) decreased lipid peroxidation and protein carbonylation in the liver, while it did not affect superoxide dismutase (SOD), glutathione (GSH), or glutathione S-transferase (GST) levels in the brain. However, CTZ significantly reduced SOD activity and increased GST activity in the liver. /The PCA revealed specific correlations, notably between SOD and iNOS, as well as between oxidative stress biomarkers and iNOS, highlighting the link between antioxidant and inflammatory responses in patients with endometriosis.
**Kalehoei E, et al. (2022) [43]**	animal study	NMRI mice	IVM (in vitro maturation) condition media	spectrophotometer analysis with a colorimetric assay, Griess reaction, fluorescence microscope	NO	NO levels significantly decreased, while TAC levels significantly increased with 0.3 and 0.6 mg/mL LC and 25 and 50% BMSC-CM compared to the control. All treatment groups showed marked improvements in IVF, cleavage, and blastocyst rates compared to the control. Additionally, there was a significant rise in the mean total cell number and TE cells with 1 mM RG, 0.3 and 0.6 mg/mL LC, and 25 and 50% BMSC-CM. /Supplementing IVM medium with LC and BMSC-CM, particularly 50% BMSC-CM, significantly boosted IVM and fertilization rates, and greatly enhanced blastocyst development and total blastocyst cell numbers in EMS-induced mice compared to the control. Overall, LC and BMSC-CM supplementation improved oocyte quality, IVM rates, and pre-implantation developmental competence post-IVF, likely by reducing nitro-oxidative stress and promoting nuclear maturation of oocytes.
**Wang D, et al. (2018) [44]**	animal study	75 Sprague–Dawley rats	endometrial tissue, peritoneal fluid	measurement of the ectopic tissues, H&E staining, immunohistochemistry, ELISA study, nitrate/nitrite kit, immunoblotting, RT-PCR	NO	Treatment with 6-shogaol significantly reduced implant size, with histological analysis showing atrophy and regression of lesions. The administration of 6-shogaol effectively down-regulated NF-κB signaling and the expression of VEGF and VEGFR-2 (Flk-1) in endometriotic lesions, while also reducing the excess production of IL-1β, IL-6, PGE2, and nitric oxide. /6-shogaol treatment significantly lowered levels of cytokines, PGE2, VEGF, COX-2, and NO, clearly demonstrating shogaol’s anti-inflammatory effects.
**Wang JH, et al. (2006) [45]**	human study	30 patients with endometriosis-associated infertility, 19 patients with carcinoma in situ of the cervix	endometrial tissue	H&E staining, western immunoblot analysis, electrochemiluminescence immunoassay	eNOS, iNOS	iNOS was not detected, while eNOS exhibited unique menstrual cycle-dependent expression. Women with endometriosis-associated infertility had higher eNOS levels in the eutopic endometrium before GnRH-a treatment compared to controls. After 3 months of GnRH-a therapy, eNOS levels decreased. Additionally, there was a significant positive correlation between serum E2 or *p* concentrations and endometrial eNOS expression. /GnRH-a treatment reduced endometrial eNOS expression in women with endometriosis-associated infertility, with endogenous ovarian steroids affecting this expression.

Abbreviation: NO, nitric oxide; NOS, nitric oxide synthase; iNOS, inducible nitric oxide synthase; eNOS, endothelial nitric oxide synthase; CTZ, clotrimazole; ELISA, enzyme-linked immunosorbent assay; SOD, superoxide dismutase; CAT, catalase; GST, glutathione-S-transferase; GSH, reduced glutathione; ADMA, asymmetric dimethylarginine; L-NMMA, L-NG-monomethyl Arginine; PCR, polymerase chain reaction; RT-PCR, real time-PCR; RFLP, restriction fragment length polymorphism; RNA, ribonucleic acid; mRNA, messenger RNA; PRR, pattern recognition receptor; PM, peritoneal macrophage; NF-κB, nuclear factor-kappa B; LPS, lipopolysaccharide; ERα, estrogen receptor alpha; ERβ, estrogen receptor beta; ERK, extracellular signal-regulated kinases; ERB-041, 7-ethenyl-2-(3-fluoro-4-hydroxy-phenyl)-1,3-benzoxazol-5-ol; HPLC, high performance liquid chromatography; MDA, malondialdehyde; IVF-ET, in vitro fertilization-embryo transfer; ADMA, asymmetric dimethylarginine; RL95-2, the human endometrial epithelial cell line; COX-2, cyclooxygenase-2; EEE, eutopic endometrium of endometriosis; NE, normal endometrium; IVM, in vitro matured; E2, estradiol; ELISA, enzyme-linked immunosorbent assay; LPS, lipopolysaccharide; PM, peritoneal macrophage; IL-6, interleukin-6; IL-12, interleukin-12; PF, peritoneal fluid; ADMA, asymmetric dimethylarginine; H&E, hematoxylin and eosin; PGE_2_, prostaglandin; E_2_, estradiol; VEGF, vascular endothelial growth factor; CPP, chronic pelvic pain; VAS, visual analogue scale; GnRH, gonadotropin-releasing hormone; P, progesterone; qPCR, quantitative polymerase chain reaction; NF-κB, nuclear factor-kappa B; EMS, endometriosis; IVM, in vitro maturation; IVF, in vitro fertilization; RG, repaglinide; LC, L-carnitine; BMSC-CM, bone marrow mesenchymal stem cell-conditioned medium; TAC, total antioxidant capacity; IFN-α, interferon-alpha; IFN-γ, interferon-gamma.

### 2.2. Studies Suggesting That NO Improves Endothelial Function in Endometriosis (Table 2)

Shoko Kinugasa reported that “increased asymmetric dimethylarginine (ADMA) and enhanced inflammation are associated with impaired vascular reactivity in women with endometriosis”. The enhanced inflammatory response, which can impair vascular reactivity, is associated with the development of endometriosis. ADMA, an inhibitor of endogenous NOS, is also linked to endothelial dysfunction. In a study involving 41 women with endometriosis and 28 without, the flow-mediated vasodilation (FMD) was significantly lower, and ADMA levels were markedly higher in women with endometriosis. Additionally, inflammatory markers were elevated in these women, indicating that increased plasma ADMA may contribute to endothelial dysfunction in endometriosis. This suggests that ADMA not only inhibits NO synthesis but also increases superoxide production, further reducing NO bioavailability [46].

Ana Filipa Martins and colleagues investigated the effects of metformin on the morphological structure, endothelial function, angiogenesis, inflammation, and oxidative pathways in the hearts of mice with surgically induced endometriosis. B6CBA/F1 mice (*n* = 37) were divided into four groups: Sham (S), Metformin (M), Endometriosis (E), and Metformin/Endometriosis (ME). Reduced eNOS expression and increased ET-1 activity are key markers of endothelial dysfunction. In the ME group, metformin treatment resulted in decreased ET-1 levels and increased eNOS expression compared to the E group. Endometriosis was associated with reduced expression of MIR199a, MIR16-1, and MIR18a. The study concluded that metformin mitigates endothelial dysfunction in endometriosis by enhancing eNOS expression [47].

Statins, known for their potent anti-inflammatory effects, have been proposed as adjunctive therapies for women with endometriosis. Gabrielle A. Dillon et al. hypothesized that impaired NO-dependent microvascular endothelial function might occur in women with endometriosis and evaluated whether short-term statin administration could improve endothelial function. In a study of eight healthy controls and eight women with endometriosis, acetylcholine (Ach)-induced vasodilation was attenuated in women with endometriosis, indicating endothelial dysfunction. NO-dependent vasodilation was also reduced. Oral atorvastatin improved Ach-induced and NO-dependent vasodilation in these women, suggesting that short-term systemic statin therapy enhances endothelial-dependent microvascular vasodilation through NO-dependent pathways in women with endometriosis [48].

In Section 2.2, this paper discussed the claim that NO has a therapeutic effect on endometriosis. This therapeutic effect is primarily thought to occur through the mediation of angiogenesis. In the previous Section 2.1, which argued that NO is involved in the pathogenesis of endometriosis, both iNOS and eNOS were equally mentioned as target pathways. However, in this chapter, only eNOS is mentioned. Additionally, there have been studies stating that ADMA inhibits vascular NO production, leading to endothelial dysfunction and vasoconstriction [46].

**Table 2:** Studies suggesting that NO plays a protective role in endometriosis.

Author [Reference]	Study Design	Subjects	Sample (s)	Detection Method	Target Pathway (s) Associate with NOS	Results/Conclusions
**Kinugasa S, et al. (2011) [46]**	human study	41 women with and 28 women without endometriosis	plasma	HPLC, high-resolution Doppler ultrasonography	NO, ADMA, SDMA	In the endometriosis group, plasma levels of inflammatory markers like hs-CRP, SAA, and IL-6, along with ADMA, were significantly elevated, whereas SDMA was not. FMD showed a significant negative correlation with ADMA levels. /ADMA, an inhibitor of NO synthesis, suppresses vascular NO production, impairing vascular reactivity, and leading to endothelial dysfunction and vasoconstriction. In this study, women with endometriosis had elevated ADMA levels, which were inversely related to FMD, suggesting a link between increased plasma ADMA and impaired endothelial function. ADMA not only inhibits NO synthase but also boosts superoxide production, further reducing NO bioavailability.
**Ana Filipa Martins (2022) [47]**	animal study	37 female B6CBA/F1 mice	cross-sectional area of cardiomyocytes, heart sections	H&E staining, picrosirius-red staining, immunofluorescence, western blotting, real-time qPCR	eNOS	In the metformin-treated endometriosis-induced mice (ME) group, metformin downregulated ET-1 and upregulated eNOS expression compared to the endometriosis-induced mice (E) group. However, metformin did not significantly reduce the elevated NF-kB expression caused by endometriosis. /Endothelial dysfunction involves reduced eNOS expression and increased ET-1 activity, with ET-1 acting as a potent vasoconstrictor and platelet activator, while decreased eNOS expression inhibits NO production. These factors contribute to endothelial damage and cardiovascular disease. In this heart study, endometriosis animals showed an antiparallel variation in ET-1 and eNOS expression compared to sham counterparts, indicating these mechanisms are active in endometriosis.
**Gabrielle A. Dillon (2022) [48]**	human study	8 healthy controls, 8 women with endometriosis		microvascular function assessment (cutaneous vascular conductance)	NO	This proof-of-concept trial shows that women with endometriosis have impaired endothelial-dependent microvascular function, partially due to reduced NO-dependent mechanisms. Seven days of oral statin improved endothelium-dependent and NO-dependent vasodilation. Our findings suggest that the impaired microvascular endothelial function in these women is partly due to decreased NO bioavailability, highlighting reductions in NO-dependent mechanisms in the microcirculation. /In conclusion, this initial proof-of-concept study indicates that short-term systemic statin treatment enhances cutaneous microvascular endothelium-dependent vasodilation in women with endometriosis by augmenting NO-dependent pathways.

Abbreviation: NO, nitric oxide; NOS, nitric oxide synthase; iNOS, inducible nitric oxide synthase; eNOS, endothelial nitric oxide synthase; ADMA, asymmetric dimethylarginine; SDMA, symmetric dimethylarginine; FMD, flow-mediated vasodilation; hs-CRP, high sensitive-C reactive protein; SAA, serum amyloid protein; IL-6, interleukin-6; qPCR, quantitative polymerase chain reaction; ET-1, endothelin-1; NF-κB, nuclear factor-kappa B.

### 2.3. Studies Suggesting That NO Is Unrelated to the Pathogenesis of Endometriosis (Table 3)

Hong-Nerng Ho investigated the role of NO and oxidative stress in the pathogenesis of adhesion formation and infertility associated with endometriosis. By examining peritoneal total antioxidant status (TAS) and NO metabolism products in women with early-stage endometriosis (*n* = 12), advanced-stage endometriosis (*n* = 12), and healthy fertile women (*n* = 10), the study found no significant differences in TAS or NO metabolites in the peritoneal fluid across these groups. Moreover, TAS and NO metabolites were not correlated with CA125, estrogen, or progesterone levels. During the early follicular phase, TAS and NO metabolite levels in the peritoneal fluids of women with endometriosis did not show significant increases. The study concluded that NO expression was not associated with endometriosis [49].

A similar outcome was observed in a study by Manjula Bhanoori, which investigated whether the eNOS gene affects the risk of endometriosis in South Indian women. The study compared single nucleotide polymorphism (SNP) Glu298Asp in exon 7 of the eNOS gene in 232 women with endometriosis and 210 healthy women. No differences were observed in genotype distributions or allele frequencies between the groups. Thus, no association was found between eNOS Glu298Asp exon 7 polymorphism and endometriosis in South Indian women [50].

However, the opinion that NO is unrelated to endometriosis is not currently gaining much agreement. Recent research trends suggest that it is more common to view NO as having either a negative or positive impact on endometriosis.

**Table 3:** Studies claiming that NO is unrelated to the pathogenesis of endometriosis.

Author [Reference]	Study Design	Subjects	Sample (s)	Detection Method	Target Pathway (s) Associate with NOS	Results/Conclusion
**Hong-Nerng Ho (1997) [49]**	human study	24 women with endometriosis, 10 fertile women without endometriosis	peritoneal fluid, serum	Griess method, measurement of total antioxidant status (TAS), Immulite OM-MA assay (to measure the concentrations of CA125, oestrogen, progesterone)	NO	There were no significant differences in TAS and NO metabolism products in the peritoneal fluids of women with early and advanced endometriosis compared to fertile women without endometriosis during the early follicular phase. TAS and NO metabolism products were unrelated to CA 125, estrogen, or progesterone levels. Serum CA 125 concentration, but not peritoneal fluid CA 125, positively correlated with endometriosis severity. /TAS and NO metabolism product concentrations did not increase in the peritoneal fluids of women with endometriosis during the early follicular phase. Further investigation is needed to understand their role in the pathophysiology of endometriosis.
**Manjula Bhanoori (2007) [50]**	human study	232 infertile women with endometriosis, 210 infertile women with no evidence of disease	endometrial tissue	PCR and sequencing analysis, immunohistochemical analysis, western blot analysis	eNOS	No statistically significant differences were found in genotype distributions and allele frequencies between cases and controls across codominant, dominant, and recessive models. The protein’s localization and expression were similar in the endometrium of both groups. /In this study, we found no difference in eNOS expression and no association between the eNOS Glu298Asp exon 7 polymorphism and endometriosis in South Indian women.

Abbreviation: NO, nitric oxide; NOS, nitric oxide synthase; eNOS, endothelial nitric oxide synthase; TAS, total antioxidant status; CA125, cancer antigen 125; PCR, polymerase chain reaction

## 3. Other Gasotransmitters (CO, H_2_S) and Endometriosis

Additionally, the impact of other gasotransmitters, namely carbon monoxide (CO) and hydrogen sulfide (H_2_S), on endometriosis was investigated. Research on these gasotransmitters is less active compared to NO, resulting in fewer related studies. However, experimental papers concluding that H_2_S exhibits a therapeutic effect on endometriosis have been identified (Table 4).

H_2_S is converted from cysteine by the enzymes cystathionine β-synthase (CBS) and cystathionine γ-lyase (CSE). Experimental results reported by Shating Lei et al. are as follows: The expression levels of CSE and CBS were highly detected by immunohistochemistry and immunocytochemistry in ectopic endometrium compared to normal endometrium. To further elucidate the effect of H_2_S on human endometrial stromal cells (HESCs) proliferation, the cell viability of HESCs was measured after the administration of NaHS. As a result, NaHS administration significantly increased the cell viability of HESCs, reaching a peak at NaHS 300 µmol/L. This pro-proliferation effect of H_2_S on HESCs was distinctly reduced by treatment with the CBS inhibitor (1 mmol/L) or the CSE inhibitor (3 and 10 mmol/L). Additionally, it was demonstrated that the NF-κB pathway could be associated with the H_2_S-induced proliferation effect on HESCs. The increased HESC viability following 300 µmol/L NaHS administration was diminished by the administration of specific inhibitors of the NF-κB pathway. These data suggest that H_2_S promotes ESC proliferation through the activation of the NF-κB pathway and could thus play a role in the pathogenesis of endometriosis [51].

Earlier, opinions were confirmed that the NF-κB pathway mediates the mechanism by which NO affects endometriosis [22,39,40,44]. Considering this relationship, the experimental results indicating that the NF-κB pathway is involved in the mechanism by which H_2_S affects endometriosis are significant.

Additionally, H_2_S has been found to impact the function of both male and female reproductive systems and may have therapeutic implications for reproductive disorders, including endometriosis. These regulatory functions of H_2_S within the reproductive systems of both genders could be related to the NO/cGMP pathway, the activation of K+ channels, and the relaxation mechanism of the spongy smooth muscle [52].

As for CO, no association with endometriosis has been identified, and no related research papers exist. However, given that NO has been shown to have some positive or negative effect on endometriosis, and H_2_S is also likely related to endometriosis, it is premature to conclude that CO is entirely unrelated to endometriosis. The need for research on this topic can be raised.

## 4. Summary

**Figure 3:**
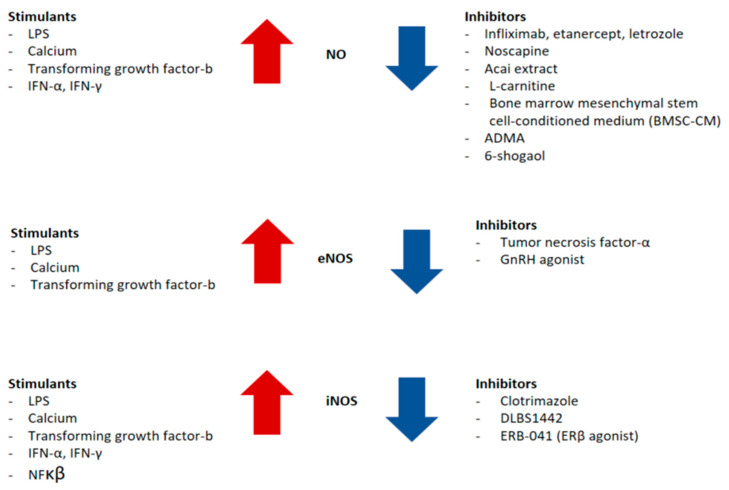
Factors influencing NO levels in endometriosis. Substances on the left of the red arrows are presumed to promote the production of NO, eNOS, and iNOS. Substances on the right of the blue arrows are presumed to inhibit their production.

NO is involved in various physiological and pathological processes, including gynecological conditions like endometriosis, affecting blood flow regulation, menstrual cycle, reproductive functions, hormone secretion, infertility, and pain. While NO is beneficial at physiological concentrations, exceeding a critical threshold can lead to toxic effects. However, the precise concentration thresholds for its physiological versus pathological roles remain unclear. Various factors influencing NO concentration have been identified in related studies, as illustrated in Figure 3.

**Figure 4:**
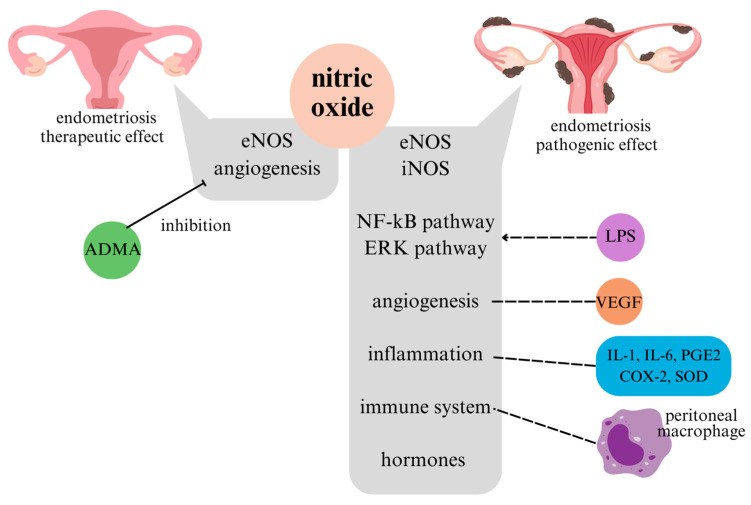
Mechanisms involved in the pathogenic/therapeutic effects of endometriosis.

In terms of the mechanism, NO involved in the pathogenesis of endometriosis can mediate pathways, such as eNOS, iNOS, the NF-κB pathway, and the ERK pathway. Additionally, angiogenesis involving VEGF, inflammatory processes involving substances like IL-1, IL-6, PGE2, COX-2, and SOD, immune responses involving peritoneal macrophages, and hormonal actions involving estrogen and progesterone contribute to the pathogenesis of endometriosis. For NO exhibiting therapeutic effects on endometriosis, it may mediate the actions of eNOS and angiogenesis. These mechanisms are illustrated in Figure 4.

## 5. Conclusions

Oxidative substances like NO exhibit dual effects: they perform physiological functions under normal conditions but can disrupt bodily functions and contribute to pathological states when balance is lost. Although the role of NO in the pathogenesis of endometriosis remains somewhat controversial, its expression is closely associated with the disease’s pathophysiology. NO levels can influence menstrual cycles, pregnancy, infertility, pelvic pain, hormones, and inflammatory factors. Conversely, these factors may also affect NO production, highlighting the complex interplay between NO and endometriosis. Therefore, the regulation of NO levels could be considered for the treatment of endometriosis.

## Figures and Tables

**Table 4:** Studies claiming that H_2_S is related to the pathogenesis of endometriosis.

Author [Reference]	Study Design	Subjects	Sample (s)	Detection Method	Target Pathway (s)	Results/Conclusion
**Shating Lei et al. (2018) [48]**	human study	samples of ectopic endometrial tissues from women with endometriosis (*n* = 21), and normal endometrial tissues from women in the control group (*n* = 35)	endometrial tissue	immunohistochemistry, immunocytochemistry, cell count kit-8 (CCK-8) assay, western blot analysis	CBS, CSE, NF-κB pathway	Both the expression of CSE and CBS were higher in ectopic endometrium from endometriosis samples compared to expression in the normal endometrium. Apparently, CBS and CSE were highly expressed in both the stroma and glandular areas. NaHS administration significantly augmented the cell viability of HESCs and reached a peak at 300 μmol/L. Additionally, we treated cells with PDTC and CAPE, which are specific inhibitors of the NF-κB pathway. Adding 300 μmol/l NaHS alone could significantly augment HESC viability, whereas the administration of PDTC diminished this effect remarkably. The same change was observed when HESCs were co-treated with 300 μmol/l NaHS and different doses of CAPE. /These data suggested that H_2_S promotes ESC proliferation via activation of the NF-κB pathway, which provides a scientific basis for the clinical application of blocking H_s_S to treat endometriosis.

Abbreviation: CCK-8 assay, cell count kit–8 assay; CBS, cystathionine β-synthase; CSE, cystathionine γ-lyase; NF-κB, nuclear factor-kappa B; HESCs, human endometrial stromal cells; PDTC, pyrrolidine dithiocarbamate; CAPE, caffeic acid phenethyl ester; H_S_S, hydrogen sulfide.
